# AI-Accelerated Discovery of Electrocatalyst Materials

**DOI:** 10.1021/acsmaterialsau.5c00135

**Published:** 2025-10-18

**Authors:** Yifan Zeng, Jun Wang, Fengwang Li, Tongliang Liu, Aoni Xu

**Affiliations:** † Sydney AI Centre, School of Computer Science, 4334 The University of Sydney, Sydney, NSW 2006, Australia; ‡ School of Chemical and Biomolecular Engineering, The University of Sydney, Sydney, NSW 2006, Australia; § ARC Centre of Excellence for Green Electrochemical Transformation of Carbon Dioxide, The University of Sydney, Sydney, NSW 2006, Australia

**Keywords:** electrocatalysis, artificial intelligence, machine learning, descriptor, computational screening, catalyst discovery, density functional theory (DFT), renewable energy

## Abstract

The rational exploration and design of high-performance, stable electrocatalysts are crucial for efficient renewable energy storage, conversion, and utilization. Artificial intelligence (AI) is revolutionizing this field by significantly reducing the time and cost associated with conventional trial-and-error experimentation and density functional theory (DFT) calculations. Advancements in data quality, computing power, and algorithms have positioned AI as a key enabler in understanding electrocatalytic mechanisms, designing advanced materials, analyzing structures, and predicting performance. This review highlights the pivotal role of AI in electrocatalyst discovery, focusing on the critical aspects of data, descriptors, and machine learning models. We discuss various AI approaches, including their applications in accelerating DFT calculations, exploring reaction mechanisms, designing electrocatalysts, and predicting performance, providing a comprehensive overview of the current state-of-the-art. We also address the challenges and opportunities in leveraging AI for electrocatalyst development, emphasizing the importance of data quality, model selection, and collaborative research. This review aims to guide researchers in effectively utilizing AI to accelerate the discovery and optimization of electrocatalysts for a renewable energy future.

## Graphical abstract

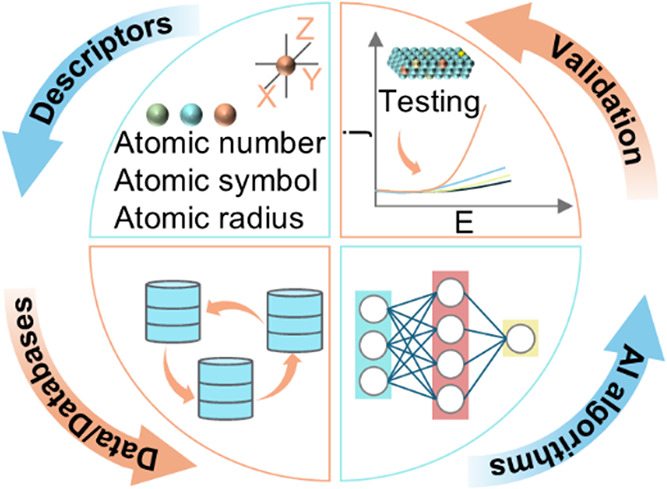

## Introduction

The demand for energy is increasing dramatically due to the growing global economy and rising population. Developing and utilizing renewable and clean energy resources, such as solar, tidal, and wind power, is a potential solution to this problem.^1^ These sources can produce clean electricity without CO_2_ emissions. For deeper penetration of renewable electricity, electrocatalysis can convert electricity into renewable hydrogen, ammonia, synthetic hydrocarbons and oxygenates, and other industrially valuable products.^2,3^ The critical issue in electrocatalysis is finding high-performance catalysts for electrocatalytic reactions to improve efficiency and reduce costs, such as lowering the overpotential to reduce electricity consumption and using nonprecious metal catalysts to reduce the capital cost of materials.^4^ The diversity of elemental combinations in electrocatalytic materials and the complexity of material structures, however, lead to long and costly development cycles for high-performance electrocatalysts.^5^

The development of electrocatalysts traditionally relies on a trial-and-error process, which can take 10–25 years or even longer to develop a material that achieves desired performance for a specific application.^4^ With advancements in computer science and hardware, computational chemistry has significantly progressed, particularly in the application of density functional theory (DFT) calculations. These developments enable deeper insights into adsorption mechanisms on catalyst surfaces, facilitating the design of high-performance electrocatalysts.^6^ However, DFT calculations are computationally expensive and resource-intensive, making it challenging to simulate large-scale surfaces and reactants precisely. Particularly in electrochemical systems, catalyst heterogeneity and environmental complexity necessitate DFT model simplifications, limiting their accuracy in describing active sites.^7^ Surface reconstruction under reaction conditions alters electronic structure and reactivity, which static DFT cannot capture.^8^ Interfacial solvent reorganization affects adsorption and reaction barriers, but explicit treatment requires extensive sampling and high computational cost.^9^ Simulating electrode potentials with charged periodic supercells often introduces artifacts due to the handling of net charges, reducing the reliability of DFT results.^10^ This limitation hinders the exploration of the vast material space and the identification of complex mechanisms.^11^

The emergence of AI has brought new possibilities to accelerate the discovery of high-performance electrocatalysts.^12^ Progress in data curation, computing power, and AI algorithms has laid a solid foundation for using AI in materials development. AI-driven methods have already been used in various fields, in particular biomedical materials and drug development.^13^ In electrocatalysis, AI-augmented DFT models can quickly predict transition state adsorption and reaction energy during catalytic turnovers.^14^ AI models can also simulate, generate, and analyze physical structure characterization data of electrocatalysts, such as X-ray Diffraction (XRD) and X-ray Absorption Fine Structure (XAFS).^15,16^ Additionally, some characteristic physicochemical descriptors of electrocatalysts can be used to quickly and accurately predict electrocatalytic properties.^16,17^

The procedure of using AI algorithms involves first defining the input data and output targets, between which a relationship is aimed to map, such as that between material structure and catalytic performance. An AI model is typically a complex function with a large number of learnable parameters, designed to approximate this mapping. These parameters are learned by fitting the training data, which consists of input-output pairs from experiments or simulations. This is known as the training procedure. After training and testing, the AI model is deployed to make predictions for the target. Although some AI models implicitly link the input and output and were traditionally viewed as a “black box”, recent advances in interpretability techniques have greatly improved this situation.^18^ Meanwhile, these models can fit the training data well and generalize to new data.

This Review aims to provide a comprehensive overview of the application of AI in electrocatalyst discovery. We first examine the pivotal role of data in AI-driven material discovery, focusing on the selection and application of appropriate descriptors. We then discuss the development of diverse AI models employed in electrocatalysis, elucidating their strengths and weaknesses, followed by a detailed exploration of how these models are applied to accelerate DFT calculations, unravel intricate reaction mechanisms, design innovative electrocatalysts, and predict their performance (Figure 1). Finally, we highlight the challenges and opportunities associated with this field, emphasizing the importance of data quality, model selection, and collaborative research. Our aim is to guide researchers in effectively utilizing AI to accelerate the development of high-performance electrocatalysts for a sustainable energy future.

**Figure 1:**
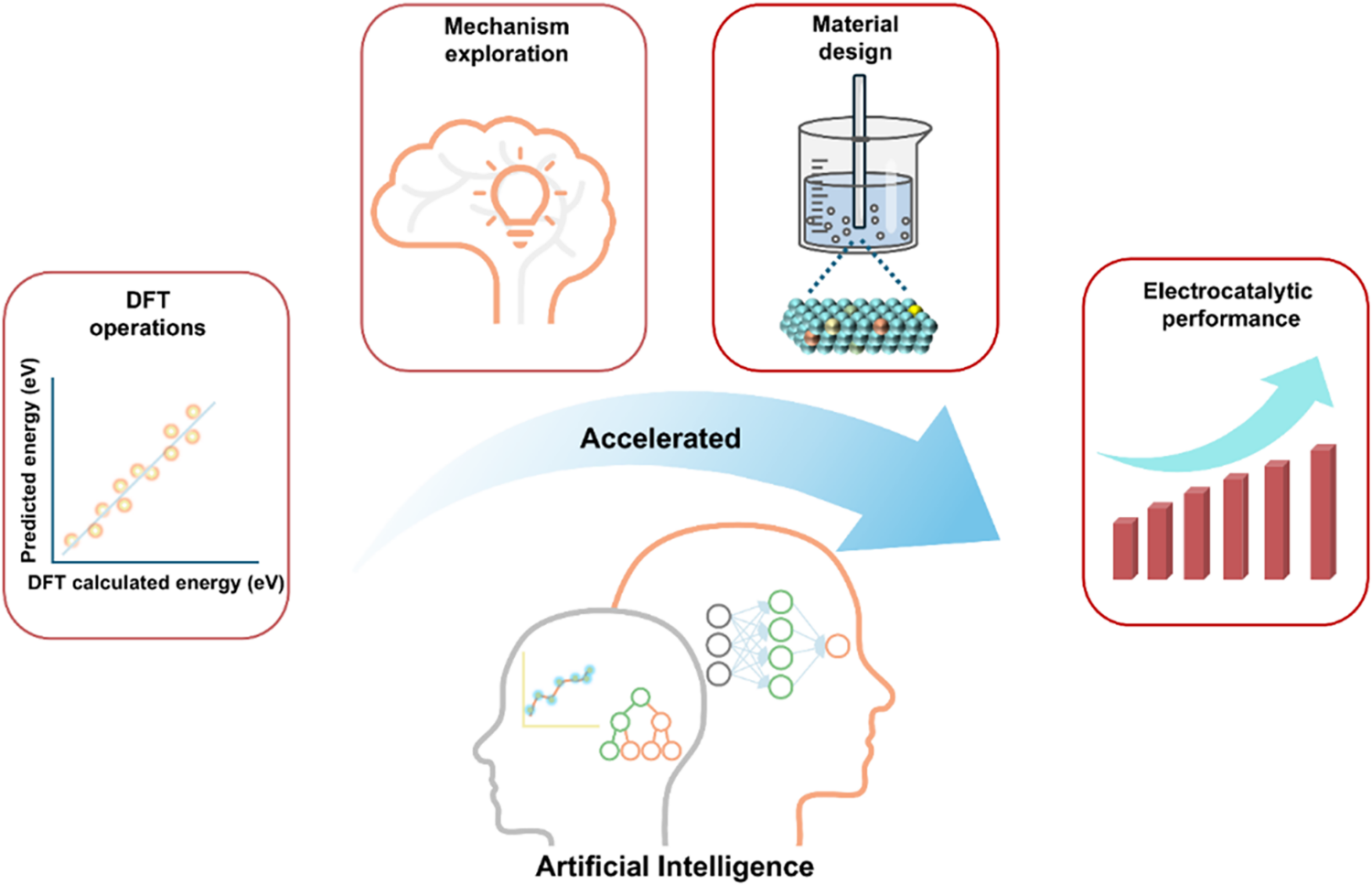
AI plays a pivotal role in electrocatalysis research for accelerating DFT calculations, exploring reaction mechanisms, designing catalysts, and predicting catalytic performance.

## Data in AI: Descriptors and Databases

Data is essential for training AI algorithms in electrocatalytic material discovery. In this section, we will review the data used in the field.

### Descriptors

Descriptors are used to characterize the physical and chemical properties of molecules, compounds, and composites in the form of vectors or tensors.^11^ By collecting appropriate descriptors as inputs and electrocatalytic performance metrics as outputs, we can train AI models to uncover the structure–activity relationships in electrocatalytic reactions.^19^ Descriptors are essential for defining the structural properties of materials. In electrocatalysis, relevant descriptors are vital for AI algorithms to make reliable decisions. These descriptors should be readily available and well-related to the electrocatalytic properties of materials.^20^

Adsorption energy descriptors measure the energy change when molecules or atoms adsorb onto a material’s surface, crucial for understanding catalytic activity and selectivity. Optimal catalytic activity occurs when intermediate adsorption is balanced. For example, hydrogen adsorption energy in the hydrogen evolution reaction (HER) and oxygen adsorption energy in the oxygen reduction reaction (ORR) are key performance indicators, often plotted in volcano diagrams.^21,22^ In the carbon dioxide reduction reaction (CO_2_RR), the CO adsorption energy influences the reduction pathway, determining whether adsorbed CO can be further reduced to hydrocarbons such as methane or ethylene.^23^ DFT studies reveal that adsorption energy depends on crystal structure, atomic distribution, and electronic properties, driving the development of descriptors such as spectral, atomic, and electronic ones. These descriptors can be used to train AI models for predicting catalyst performance and reaction pathways.

Spectra-based descriptors are derived from analyzing spectral data of a material. They provide valuable information about the material’s structure and composition. Among these, descriptors based on spectroscopic characterization, such as XRD,^16^ XPS,^24^ XAFS,^15^ Raman Spectroscopy,^25^ and IR^26^ play an essential role. XRD has been used as a descriptor of oxygen evolution reaction (OER) activity of perovskite oxide.^16^ XAFS was used to track chemical and structural changes on the catalyst’s active sites for the CO_2_RR, providing insights into its composition and correlations with catalytic activity and selectivity.^27^

Atomic structure descriptors characterize atomic-level information. They modulate adsorption energy by altering the atomic environment on the catalyst surface, including atomic arrangements and interatomic interactions. Atomic structure descriptors include atomic radius, number of protons and electrons, mass, crystal surface structure, lattice parameters, surface defects, active sites, and adsorption sites. All these features are closely related to the electrocatalytic activity.^11,28,29^ For instance, Pt(100) crystal surfaces are proved to be the most electrocatalytically active crystal surfaces in the ammonia oxidation reaction (AOR);^30^ Reasonable modulation of oxygen vacancies on the surface of OER catalysts promotes OER activity.^31^

Electronic descriptors quantify the electronic properties of a catalyst. By tuning the electronic structure parameters, the adsorption energy of reaction intermediates can be modulated, influencing electrocatalytic properties. These descriptors typically include valence electrons,^32^ Fermi level,^33^ band gap,^34^ electronegativity,^4^ ionic Lewis acid strength (ISA),^35^ highest occupied molecular orbital,^36^ lowest unoccupied molecular orbital,^36^ d-band center (ε_d_),^37^ and orbital hybridization.^38^ Narrowing the band gaps shifts the absorbance band to lower energies, enhancing HER performance,^34^ the *e*_g_ orbitals of surface transition metals engage directly in σ-bonding with surface-adsorbed species, influencing their bond strength and tuning OER activity,^38^ and ε_d_ can serve as an effective descriptor for ORR, CO_2_RR, and nitrogen reduction reaction (NRR).^35,37^

Taken together, these examples illustrate a natural hierarchy: atomic structure descriptors capture fundamental features, electronic and adsorption energy descriptors represent material properties, while spectra-based descriptors reflect experimental measurements. These descriptors are typically discussed individually for correlating electrocatalytic activity in research articles. However, electrocatalytic reactions are often highly complex, making it challenging to directly predict electrocatalytic performance based merely on one specific descriptor. To address this, symbolic regression techniques such as sure independence screening and sparsifying operator (SISSO) have proven highly effective in constructing more informative descriptors.^39^ By systematically applying mathematical operators and compressed sensing, SISSO can automatically generate low-dimensional, physically meaningful composite descriptors from vast and correlated feature spaces, even when only limited data is available. For example, SISSO has been applied to identify stability descriptors for acid-stable oxide electrocatalysts^40^ and to capture charge-transfer related descriptors in single-atom catalysts on ceria,^41^ illustrating its ability to derive meaningful, task-specific descriptors for electrocatalysis. This greatly enhances model interpretability and facilitates the discovery of hidden structure–property relationships, offering a promising pathway to advancing AI-driven materials discovery. AI introduces potential improvement by training prediction models on various descriptors for a range of reaction conditions.

### Databases

Following the definition of descriptors, databases provide the structured data required to train and validate AI models, and such data lies at the core of AI-driven electrocatalyst discovery. There are three primary sources of material descriptor data: experimental data, such as XRD and XPS,^24^ theoretical data, such as adsorption energies and electron spin energies,^42^ and data obtained from established databases, which include both experimental data and theoretical models from researchers.^14^ For researchers, obtaining descriptor data directly from databases is the most direct and convenient way, which saves time on repeating experiments and calculations and allows researchers to concentrate more on improving AI models. The most comprehensive databases include Materials Project, Open Quantum Materials Database (OQMD), AFLOW for materials discovery, the Novel Materials Discovery (NoMaD) repository, and ElectroCat Database.^11,43,44^ In addition, data sets such as Alexandria^45^ and the joint automated repository for various integrated simulations (JARVIS)^46^ are widely used for training machine learning interatomic potentials, with Alexandria providing high-throughput quantum chemistry data and JARVIS, developed by NIST, offering extensive DFT, molecular dynamics, and experimental benchmarks. More recently, the Digital Catalysis Platform (DigCat) has been introduced as the largest electrocatalysis database, integrating extensive experimental performance records with AI-powered tools to support catalyst discovery.^47^ Researchers can search them for thermodynamics, spectra, and electronic structures. The Open Catalyst Database, developed by Meta and Carnegie Mellon University, marks a major step forward in applying machine learning (ML) to heterogeneous catalysis. It supports accurate, generalizable models for predicting catalyst properties and reaction pathways, while minimizing the reliance on expensive DFT computations. Open data sets, baseline models, and leaderboards further promote community development.^48^ Furthermore, the ElectroCat Database provides experimental electrochemical performance data, which are particularly valuable for benchmarking catalyst activity and stability. Building on both computational and experimental resources, the Open Catalyst Experiments 2024 (OCx24) data set integrates high-throughput experimental data, including gas diffusion electrode tests for HER and CO_2_RR activities, with large-scale computed adsorption energies.^49^ This enables the training of models that directly link theoretical descriptors to experimental performance, thereby improving the applicability and reliability of AI-driven catalyst design.

While these databases provide a wealth of trainable data for AI model training and researchers can contribute data to further accelerate catalyst design and optimization processes, challenges remain, such as the need for more standardized data management. Due to variations in experimental protocols, such as catalyst loadings, electrolytes recipes, and testing temperatures, electrocatalyst data are not standardized, making it difficult to train AI models for performance prediction. Additionally, data in many databases tend to be filtered, with researchers reporting only data that align with expectations, while discarding data that do not contribute to the conclusions. However, discarded data are necessary for modeling complete data distributions and ensuring the model’s generalization performance, which improves the model’s overall performance and predictive ability to cope with unknown data.

## AI Models for Electrocatalysis

With descriptors and databases in place, AI models can then be employed to uncover structure–property relationships. In practice, they correlate catalyst physicochemical properties as input with electrocatalytic performance as output. Given the same inputs and outputs, different AI models can, however, describe different relationships. Since the closed-form relationship is *apriori* unknown, different AI models function as different approximations to the target relationship. Designing a powerful AI model is important to improve the prediction reliability of AI algorithms. AI models and their optimization methods (to learn the parameters of the model) have undergone decades of development. Selecting the appropriate AI model according to the complexity of the problem and the characteristics of the data is crucial for enhancing predictive accuracy.

Multivariate linear regression (MLR) is a classic and one of the simplest algorithms in AI modeling (Figure 2A). It is mainly used to establish a linear relationship between the independent variables (features) and the dependent variable (target). Its advantages lie in computational efficiency, simplicity, and interpretability. It is especially suitable for small data sets and linear input-output relationships. In the study of ORR, combining correlation and linear regression analyses confirm that the oxygen affinity of the carrier metal plays a vital role in both activity and stability.^50^ However, reaction mechanisms can be complex. Sometimes, finding a linear correlation between input descriptors and output performance is difficult and introduces approximation errors.

**Figure 2:**
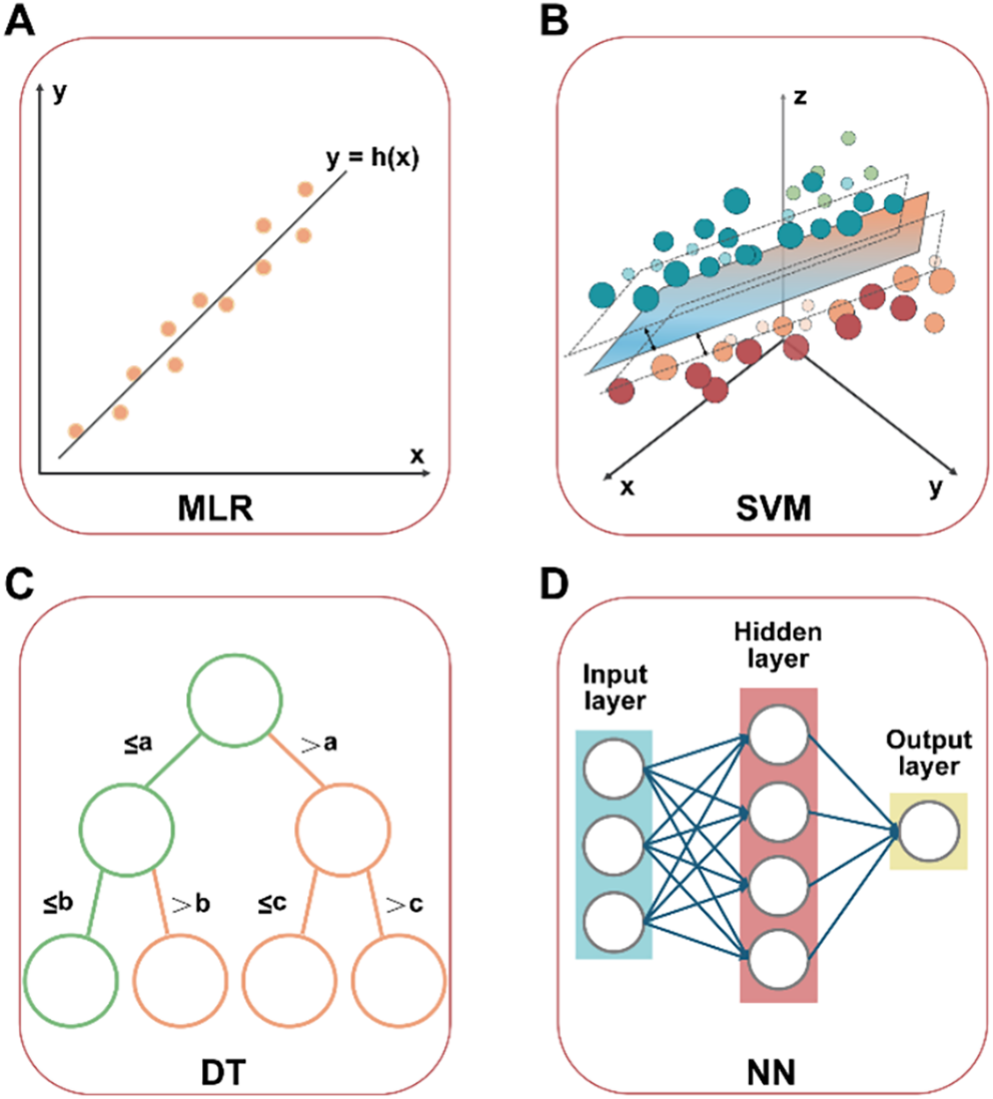
Typical algorithms used in AI for electrocatalysis. (A) Multivariate linear regression, (B) support vector machines, (C) decision trees, and (D) neural networks.

To address this, support vector machines (SVMs) are gradually being utilized in materials research (Figure 2B). When data points are not linearly separable, SVMs avoid manual feature mapping. Instead, they use kernel functions to transfer data into high-dimensional latent space, providing a nonlinear solution through high-dimensional linearity. SVMs suit classification problems with small data sets and high-dimensional spaces. In CO_2_RR, support vector regression (SVR), an extension of SVM, accurately predicted CO_2_RR properties by combining descriptive descriptors.^51^ Using simple features, SVR can also predict the Gibbs free energy of hydrogen adsorption (Δ*G*_H*_) in HER studies.^52^ However, with large, noisy data sets, SVMs struggle to fully utilize information, and kernel functions can cause memory and time overhead, making them inefficient.

For medium-sized data sets, decision trees (DTs), random forests, and boosting algorithms based on DTs offer accurate results quickly. They are a common choice in many applications. DTs are tree structures where each internal node represents an examination on a feature, each branch represents an examination result, and each leaf node represents an output (Figure 2C). DTs are fast to train, accurate, and easier to interpret than alternative AI methods.^53^ Random forests and boosting algorithms utilize ensemble learning to train many DTs simultaneously and synthesize their results to improve accuracy further. In HER studies of transition metal (TM) phosphides, tree-based algorithms have accurately predicted Δ*G*_H*_.^54^ DTs are also powerful tools for guiding experimental studies. They have good regression capabilities for ammonia productivity and faradaic efficiency in NRR and help extract feature significance.^55^ However, DTs can be unstable, with small data changes leading to different tree structures. Feature correlations may be ignored due to local optimality in feature selection. Additionally, DTs lack feature processing capabilities, making it challenging to extract high-level semantics from raw features, limiting their performance in high-dimensional big data.

With the increasing availability of data, neural networks (NNs) are now widely applied, particularly for handling complex nonlinear relationships and large-scale data sets. These capabilities enable researchers to identify structure–activity relationships with higher accuracy. NNs consist of multiple neurons and layers that can be trained to represent complex nonlinear relationships through activation functions and weight connections (Figure 2D). NNs have high expressive power.^56^ With vast data sets, NNs can efficiently capture complex structures and patterns, have good generalization ability and make accurate predictions for unseen data–with proper training and tuning. Different types of NNs have been used in electrocatalysis studies. For example, a neural network language model (NNLM) was used to correlate atomic environment with formation energy of catalyst as well as the free energy of OER intermediates, resulting in the discovery of an optimized catalyst with low theoretical overpotentials.^57^ In some cases, even a small, fully connected NNs can be effective enough, such as predicting the TM dual-atom catalysts on N-doped graphene support for HER performance.^58^ NNs were considered as a black-box model with limited interpretability of their prediction results and internal decision-making processes, which may be undesirable for some applications. Nevertheless, recent advances have progressively improved their transparency. Techniques such as Shapley additive explanations (SHAP)^59^ and local interpretable model-agnostic explanations (LIME)^60^ quantify the influence of input features on predictions, enhancing interpretability. In practice, these approaches are often most effective when combined with traditional descriptors, which helps ensure that the resulting interpretations remain physically meaningful. Symbolic regression distills learned relationships into analytical expressions, bridging data-driven models with theoretical understanding.^40^ Physics-informed and theory-infused neural networks (TinNet) incorporate physical laws and domain knowledge directly into model architectures, ensuring outputs remain consistent with scientific principles.^61^ Bayesian statistical learning methods further enhance interpretability by introducing uncertainty quantification.^62^ These developments are gradually transforming NNs into more transparent, scientifically insightful tools with high predictive power.^63^

In addition to classical ML models widely used in electrocatalysis, modern architectures such as generative models and multimodal learning frameworks have shown great promise in inverse materials design and complex system modeling. Variational Autoencoders (VAE) are representative generative models capable of compressing high-dimensional structural data while decoding new structures from learned latent spaces^64^ (Figure 3A). In CO_2_RR, a generative framework integrated VAE with graph neural networks (GNN) to develop the crystal diffusion variational autoencoder model (CDVAE), enabling inverse design of alloy catalysts. Generated candidates were optimized via particle swarm algorithms, with alloys such as CuAl and Sn_2_Pd_5_ achieving approximately 90% Faradaic efficiency.^65^ Diffusion models (DM) are deep generative approaches that produce physically meaningful structures through iterative perturbation and reverse denoising processes^66^ (Figure 3B). Within the CDVAE architecture, diffusion processes were integrated with GNN for crystal structure generation and performance optimization.^67^ Compared with conventional VAE, DM offers improved structural diversity, fidelity, and convergence stability. Flow-matching systems such as flow matching for materials (FlowMM), further enhance the efficiency of generating complex crystal structures by learning time-dependent vector fields that convert the generative process into a deterministic trajectory, thereby significantly reducing sampling costs.^68^ On the MP-20 data set, FlowMM demonstrated over a 3-fold increase in generation speed compared to DM, while producing electrocatalyst structures with high stability.^69^ To handle heterogeneous inputs such as images, spectra, and electrochemical data, multimodal models integrate structured and unstructured sources to capture complex structure–performance–processing relationships (Figure 3C). For instance, a multimodal model combining optical microscopy images and performance data accurately predicted fuel cell performance levels, achieving a high regression accuracy (*R* ^2^ = 0.83) and identifying optimal operating conditions such as temperature and Pt loading.^70^ Together, these AI approaches enhance both predictive accuracy and generative capacity, offering promising tools for electrocatalyst design and interpretation.

**Figure 3:**
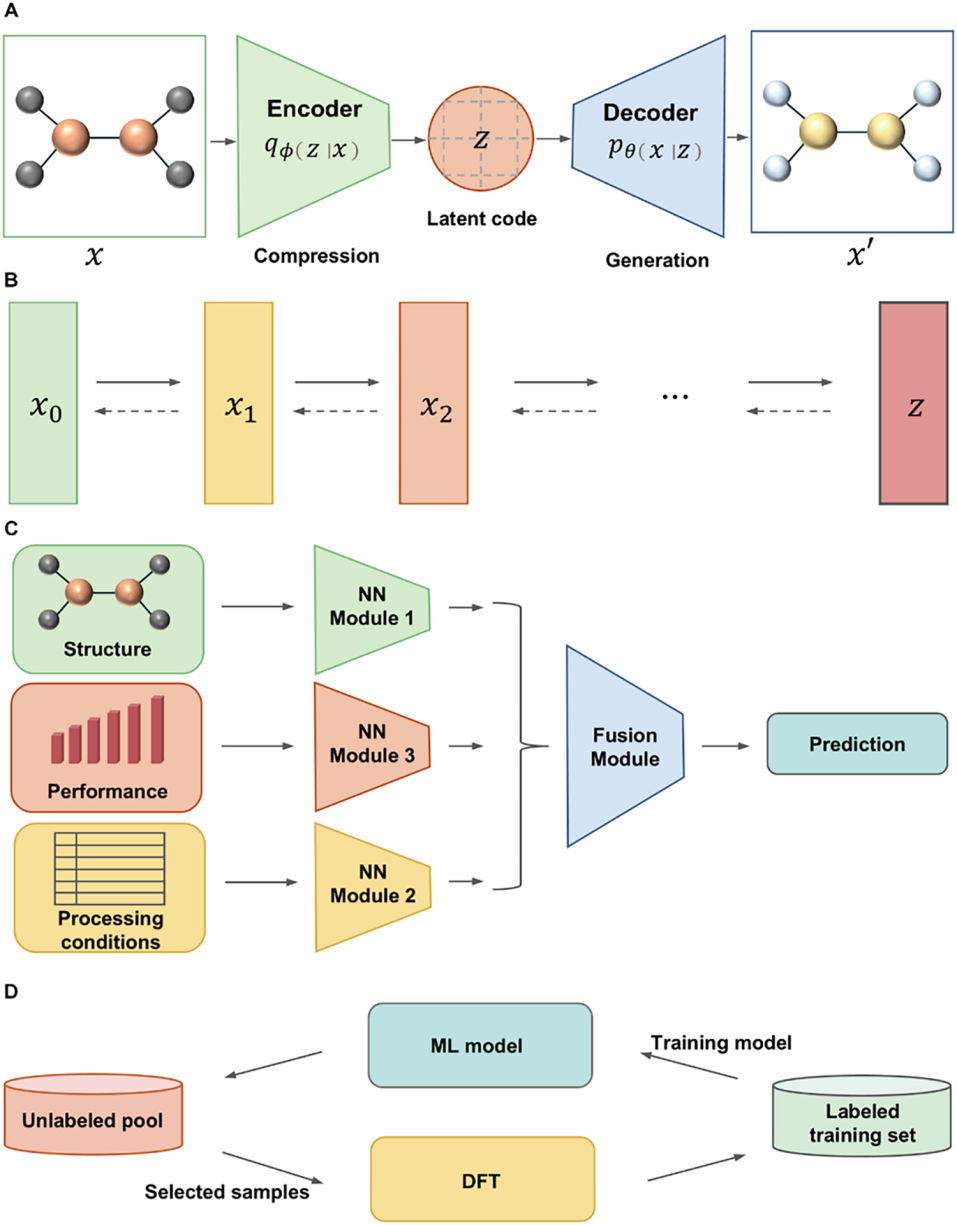
Workflows for different modern architectures and AL. (A) VAEs, (B) diffusion models, (C) multimodal models, (D) AL.

Recently, large language models (LLM) offer new opportunities to lower the barrier for chemistry researchers to engage with AI tools. LLM allows intuitive access to data analysis, property prediction, and hypothesis generation without the need for programming or ML expertise. For example, LLM have been successfully applied to literature mining and structured data set construction for CO_2_RR catalysts^71^ while language models trained on text data can predict adsorption energies with accuracy comparable to GNN.^71,72^ In addition, generative frameworks have been employed to assist in reaction condition optimization, enabling iterative improvements through natural language instructions and function-calling capabilities.^73^ These advances suggest that LLM are gradually transitioning from a purely computational tool to an important collaborative partner in scientific research.

Beyond selecting appropriate models, optimization algorithms, and improvements (e.g., incorporating prior knowledge) based on the problem environment are key to increasing AI accuracy and efficiency. The optimization algorithm uses data sets to learn model parameters during training. First-order optimization algorithms, such as stochastic gradient descent, dominate this field.^74^ They use gradient information to indicate the direction of iteration. Some modifications, such as dynamic learning rates and momentum, can enhance stability and efficiency while mitigating local minima issues.^75^ Second-order optimization algorithms can leverage the Hessian matrix for faster convergence when dealing with small data sets.^76^ However, these methods have stricter requirements for the objective and may be computationally complex with larger data sets. In the absence of gradient information, zeroth-order optimization algorithms, such as various genetic algorithms, can optimize with the values of the objective only.^77^ Although their speed may be slower without the guidance of the gradient, they are commonly used for complex, expensive-to-evaluate, or nondifferentiable objective functions. Among them, Bayesian optimization (BO) offers a powerful zeroth-order strategy for guiding experimental exploration. BO employs surrogate models, typically Gaussian processes, combined with acquisition functions to balance exploitation and exploration, allowing researchers to strategically identify the most promising candidates while minimizing expensive evaluations. Recent studies have demonstrated its effectiveness in electrocatalysis, where the vast compositional space and high evaluation costs pose significant challenges.^78^ For example, BO has been used to efficiently optimize multimetallic alloy compositions for ORR catalysts, identifying top-performing compositions with 6 to 7 times fewer evaluations compared to exhaustive grid searches.^79^ Similarly, BO-guided workflows have accelerated the discovery of stable Ir–Mo oxide electrocatalysts for acidic OER by about 8 times, achieving reduced Ir dissolution and improved activity compared to pure IrO_*x*_ controls.^80^ These examples illustrate BO’s ability to rapidly navigate complex chemical spaces and prioritize experimental efforts. Besides, adjustments and improvements can be introduced to the learning process according to specific problem environments. For example, noise is unavoidable in the results of both chemical experiments and DFT calculations. Methods such as sample reweighting can reduce the negative effect of noise.^81^ If researchers can control the data sampling process, such as deciding which experiments to conduct, AI can estimate the importance of unknown samples, thereby reducing the cost of labeling using active learning (AL).^82^ In this context, AL has emerged as a powerful strategy for catalyst screening (Figure 3D). Its key advantage is that it minimizes the number of required calculations or experiments by focusing on the most informative samples. A representative framework combining volcano plot analysis, automated ML, and ensemble regression models enables efficient identification of intermetallic compounds with near-optimal adsorption energies through iterative prediction and uncertainty-driven sampling, substantially reducing DFT computation costs.^83^ This framework has been extended to CO_2_ reduction, where a closed-loop workflow integrating DFT, ML, and in situ characterization has enabled effective mapping from structure to catalytic performance in Cu-based alloys.^14^ With continued methodological advances, NNs have been incorporated into AL workflows. For instance, combining GNN with AL has been applied to predict OER activity from XRD spectra. To address further challenges such as limited data and complex reaction pathways, approaches like transfer learning and multilabel learning have been adopted. Transfer learning leverages knowledge from related tasks to improve generalization,^84^ while multilabel learning captures interdependent reaction mechanisms, enabling more comprehensive and interpretable predictions.

Holistically, improving the predictive performance of AI algorithms in electrocatalyst discovery is a systematic task. It requires selecting informative descriptors, providing sufficient high-quality training data, using appropriate learning models and optimization algorithms, and making necessary improvements according to the task environment.^51^ The choice of AI model should depend on the specific research question. As the amount of data and complexity of features increases, overly simple models may fail to capture complex relationships. Conversely, excessively complex models can easily overfit simple data, performing well on the training set but poorly on unseen examples.

## Examples of AI Accelerated Electrocatalyst Discovery

AI has been successfully applied to a wide range of problems in electrocatalysis. It accelerates the DFT process, enabling rapid identification of optimal adsorption energies. It also speeds up the understanding of electrocatalytic mechanisms, facilitates catalyst design and characterization, as well as improves performance prediction. In this section, by highlighting these successful applications, we demonstrate not only the transformative impact of AI but also its current limitations, such as data scarcity, lack of generalizability, and limited interpretability.

### Acceleration of DFT Calculations

This subsection focuses on how AI can accelerate DFT computations and reduce the cost of modeling catalyst energetics and reaction pathways. DFT is not suitable for large-scale model calculations, and, as a result, DFT studies often rely on oversimplified models, omitting significant details and depending on researchers’ expertise to streamline them effectively. The complexity of catalyst structures and diverse element ratios make DFT struggle to meet the extensive computational demands for these tasks. Combining AI and DFT calculations can address this challenge, where AI approximates DFT calculations by reducing computation time significantly. Many ML models have been developed to approximate DFT, ranging from linear regression,^85^ Gaussian processes,^86,87^ and shallow NNs^88,89^ to more sophisticated GNN^90–92^ and Transformers.^93,94^ In general, simpler models require less training data and offer faster computation but may struggle with complicated systems. In contrast, more complex models often demand larger data sets and incur higher computational costs while better handling chemical complexity. Notably, pretrained universal models can significantly alleviate the user’s data collection burden for complex models through transfer learning.^90,91,95^ Furthermore, while most ML approaches excel at short-range interactions, recent efforts have focused on models that approximate long-range forces such as electrostatics and the effect of electric fields.^96–99^ Combining long and short-range models may improve overall accuracy. From an application aspect, this approach has been demonstrated in screening efficient single-atom OER catalysts. The physical properties and OER performance of nonradioactive TM in graphene-supported single-atom catalysts (SACs) were investigated by training a topological information-based AI algorithm.^100^ This algorithm used the results of a small number of partial DFT calculations of the TM in the preliminary stage as inputs, achieving a speedup of 130,000 times compared to using DFT calculations alone (Figure 4A). Besides, combining high-throughput DFT and GNN enables the accurate prediction of free energy change of H (Δ*G*_H*_) and CO (Δ*G*_CO*_) in 17,000 boron-doped graphene double-atom catalysts (DACs) systems.^101^ This enables the successful prediction of electrocatalysts with high HER or CO_2_RR activity. The time required to calculate DACs using AI prediction and high-throughput screening instead of time-consuming DFT was reduced significantly by a factor of about 38,400 (Figure 4B).

**Figure 4:**
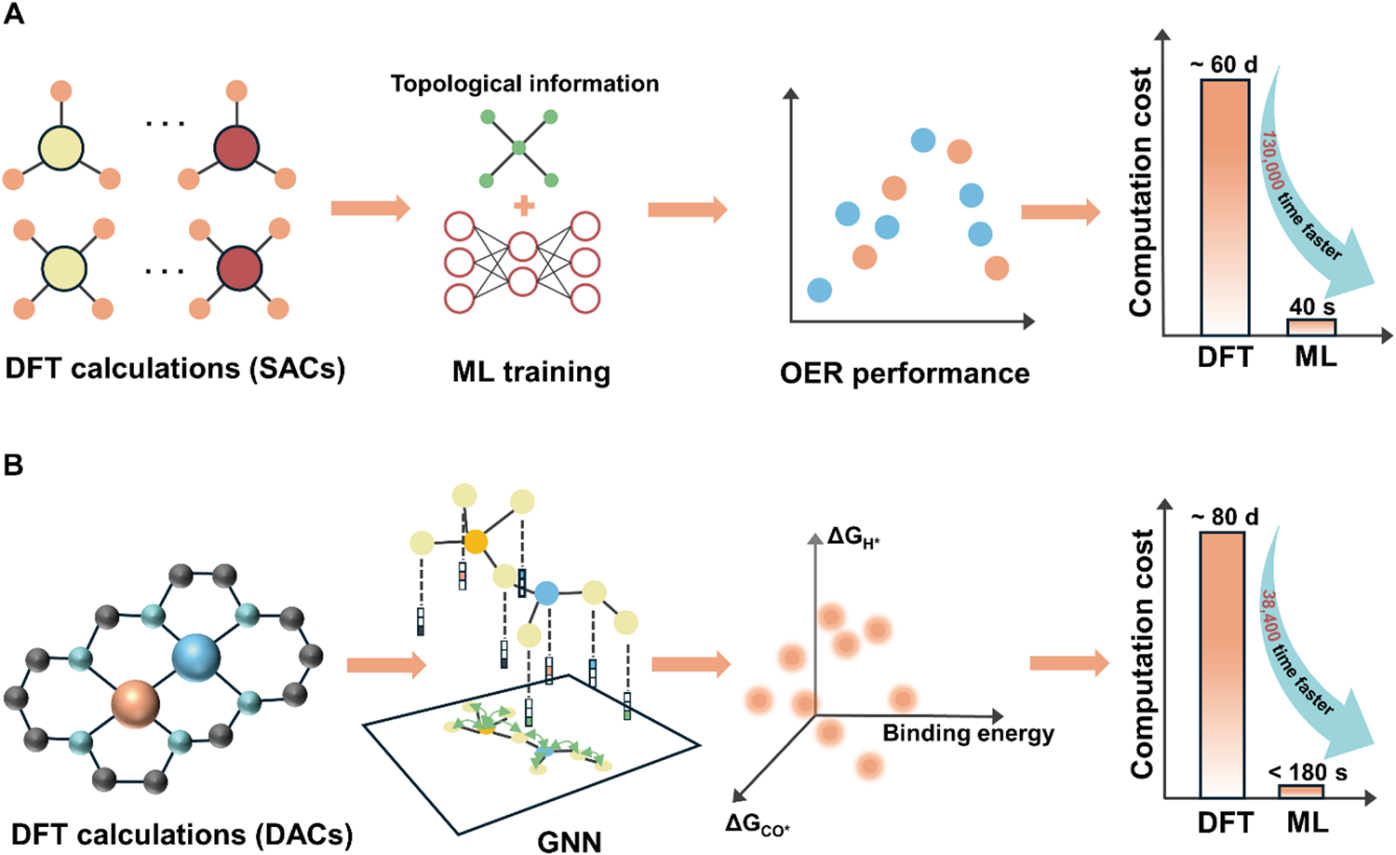
Workflows for ML to accelerate DFT calculations. Screening of (A) high-performance OER catalysts in SACs and (B) high-performance HER and CO_2_RR catalysts in DACs. (A) Reproduced with permission from ref (^100^). Copyright 2021 Elsevier.

In the exploration of PtIr-X ternary alloys for high-performance AOR, with the increase in number of possible elemental species and the determination of adsorption sites and adsorption states, the number of possible models constructed on trimetallic nanocatalysts terminating on the (100) surface exceeds 24,000 species, which is too time-consuming to be calculated only by DFT. To address this, a theory-infused neural network (TinNet, Figure 5A–C) was trained by randomly sampling 500 catalyst structures from 24,000 samples and calculating their N binding energies (Δ*E*_N*_) by DFT.^102^ The model is then optimized in conjunction with AL until convergence. Finally, AOR performance volcano diagrams were generated. The volcano diagrams screened ternary PtRu-M (M: Co, Ni, or Fe) catalysts with high AOR activity (Figure 5D). This method dramatically reduces the computational time for DFT prediction of Δ*E*_N*_, speeding up discovery and optimization of AOR electrocatalysts.

**Figure 5:**
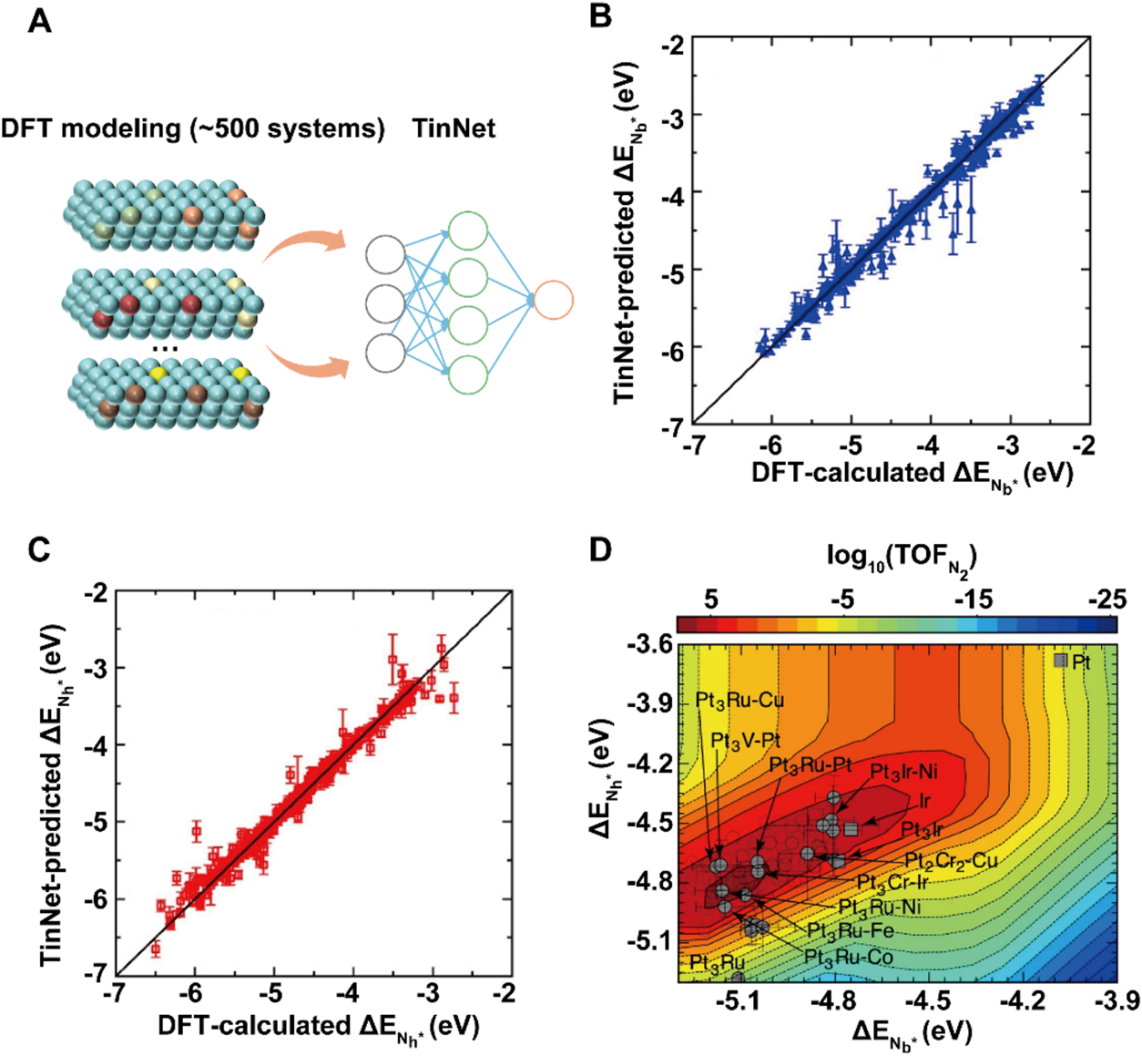
Screening of ternary PtIr-M alloys for AOR with TinNet. (A) The workflow of TinNet. (B–C) Comparison of predicted and DFT-calculated adsorption energies of N bridges (Δ*E*_Nb*_) and hollow species (Δ*E*_Nh*_). (D) The AOR activity map showing promising ternary alloy electrocatalysts predicted from the workflow. Reproduced from ref (^102^). Available under a CC-BY 4.0 license. Copyright Pillai et al.

### Acceleration of Mechanism Exploration and Characterization Data Analysis

Here we highlight the use of AI to directly predict electrocatalyst performance. Exploring the mechanism of electrocatalysts is challenging due to the inherent complexity of electrocatalytic reactions, such as multiphase interfacial reactions, complex intermediates, transition states, and different reaction pathways. Currently, the mainstream research method is still DFT calculations. The emergence of AI has brought a new direction for exploring electrocatalytic mechanisms. Through the interpretability of AI algorithms, researchers can analyze the importance of different features in the input data to understand the reason for improving catalytic material performance, thus gaining insights into the reaction mechanism.

In multicomponent systems, the complex geometrical and electronic interactions of the collective effect inside the catalyst are difficult to explain by experimental characterization alone, which hinders mechanistic understanding. Combining with AI offers a viable solution to this problem. For example, in the ORR, the ε_d_, work function (*W*_f_), magnetic moment (μ_s_), the amount of transferred charge (*Q*), and the bond length (*d*) descriptors can be used to train the gradient-boosted regression algorithm (GBR) to obtain a high-precision ORR overpotential (η_ORR_) prediction model.^103^ A weighting analysis can then be performed to derive the degree of influence of each descriptor on the ORR response. This approach was used to analyze the multicomponent catalyst Fe_SAs+NPs_Ce_SAs+Fe‑ONPs_/NC, revealing that the ε_d_ has the most significant influence on the catalytic activity (Figure 6A).

**Figure 6:**
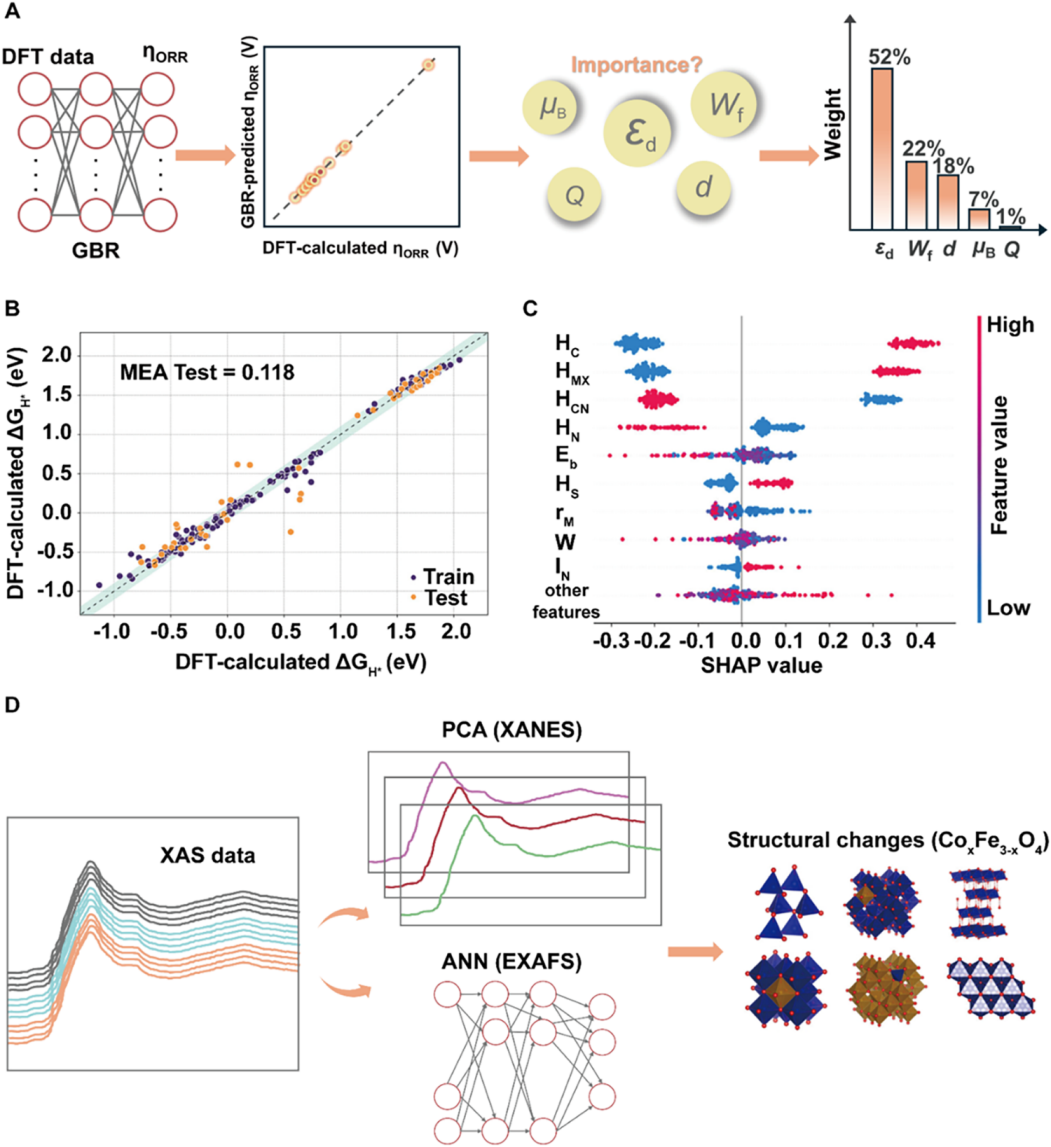
Accelerating catalytic mechanism understanding and data analysis. (A) Representation of GBR for analyzing the importance of descriptors in ORR. (B) Comparison of predicted and DFT-calculated Δ*G*_H*_ in HER. (C) The impact of each feature using the SHAP in HER. (D) Schematic of using PCA and ANN to accelerate XAS data analysis for OER mechanistic studies. (A) Reproduced with permission from ref (^103^) Copyright 2024 Wiley-VCH. (B, C) Reproduced with permission from ref (^59^). Copyright 2024 American Chemical Society. (D) Reproduced with permission from ref (^15^). Copyright 2023 American Chemical Society.

SHAP is a method for interpreting the predictions of ML models. It quantifies the impact of each feature on the final prediction by decomposing the contribution of the model prediction into the contribution of each feature value. In a study of HER performance of heterostructures with TM atoms inserted into g-C_3_N_4_/MX_2_ (M = Mo, W; X = S, Se, Te) insertions, a high-accuracy prediction model was trained by random forest regression (Figure 6B).^59^ The subsequent analysis by SHAP revealed that Δ*G*_H*_ on the C site, MX layer, S site, and intercalation of TM atoms at the N site all critically affect the HER performance (Figure 6C). However, in the AOR reaction, local interpretation of Pt_3_Ru relative to Pt and Pt_3_Ru_1/2_Co_1/2_ relative to Pt_3_Ru shows that the adsorbate resonance energy of the N_2p_ frontier orbitals is the most critical factor controlling site catalytic performance, a finding different from traditional view that the ε_d_ is a prominent factor affecting catalyst performance.^102^ Additionally, AI has the capability to analyze characterization data fast and accurately. PCA was used to understand the subtle changes in the XANES for studying OER mechanism (Figure 6D).^15^ An artificial neural network (ANN) was developed to decipher the EXAFS. Then, by correlating the structural changes with the different catalytic properties of a series of Co_*x*_Fe_3–*x*_O_4_ samples with varied catalytic activities, this approach elucidates the structural evolution of this catalyst, which helps reveal the OER mechanism.

Beyond catalytic descriptor, a deeper insight into mechanism would benefit a broad field of electrochemical systems and require further expanding the scope of AI interpretability. Interpretable AI models, such as TinNet,^18^ are capable of identifying key descriptors associated with catalytic performance and elucidating how specific physicochemical properties, such as the adsorbate resonance energies of frontier orbitals and the interatomic coupling coefficient, influence reaction pathways, including adsorption, bond dissociation, and intermediate formation.^102^ By expanding beyond static descriptor identification, AI can assist mechanistic understanding from two complementary perspectives: tracking surface intermediates that govern catalytic behavior and capturing material evolution under realistic operating conditions. On one hand, by analyzing different adsorption states and reaction environments, interpretable AI models can reveal how the importance of physicochemical features evolves, thereby facilitating the understanding of catalytic behavior under realistic operating conditions. On the other hand, by combining AI with analysis of characterization data, it is possible to uncover subtle variations in catalyst structures that are difficult to detect through traditional methods, thereby accelerating the interpretation of complex structural features and improving the accuracy and efficiency of mechanistic understanding.^15^ Based on this, integrating AI-based interpretability with structural characterization enables the tracking of intermediate species evolution and the elucidation of active site dynamics and deactivation mechanisms.^63^ This integrated approach effectively bridges the gap between the static structure-performance relationships derived from initial physicochemical descriptors and the dynamic structural changes of catalysts under operating conditions, leading to a more realistic and comprehensive understanding of electrocatalytic systems. By combining descriptor-based interpretability analysis with mechanistic studies driven by characterization data, a multidimensional and organized framework for catalyst discovery can be established. This comprehensive strategy not only accelerates the identification of key descriptors and active sites but also deepens the fundamental understanding of reaction mechanisms, ultimately promoting the rational and sustainable design of catalysts.

### Accelerate the Design of Material Compositions and Structures

This part highlights how AI supports the efficient exploration of catalyst compositions and structures. AI-guided exploration of catalyst structures can guide the design of catalyst components and structures, drastically reducing time for catalyst screening and development. In an example of designing high-temperature ORR catalysts, computational costs were reduced and efficiency was improved by using only molecular formulas without building molecular models.^35^ ISA values, ionic electronegativities, ionic radii, and ionization energies, as well as the tolerance factor, were chosen as descriptors to train an ANN, obtaining highly accurate prediction models. Based on these results, four highly active ORR catalysts were screened from 6871 chalcogenide compositions.

Advances in AI-related hardware and software have led to fully automated, high-throughput chemical experimental robots that operate without human intervention, which further accelerates the discovery of high-performance electrocatalysts.^104^ Given five Martian ore raw materials as the source of OER electrocatalytic materials, researchers can deduce more than 3.76 million catalyst formulas.^105,106^ It would take 2000 years to test these using manual experimentation. Researchers selected 6-metal OER catalysts for the study and then used a fully automated AI chemist robot (Figure 7A) to learn from more than 30,000 theoretical data and 243 experimental data in 6 weeks. This successfully trained the OER prediction model through the ANN method and screened out the catalyst with optimal composition capable of efficiently and stably operating at −37 °C on Mars (Figure 7B,C), significantly reducing catalyst screening time.

**Figure 7:**
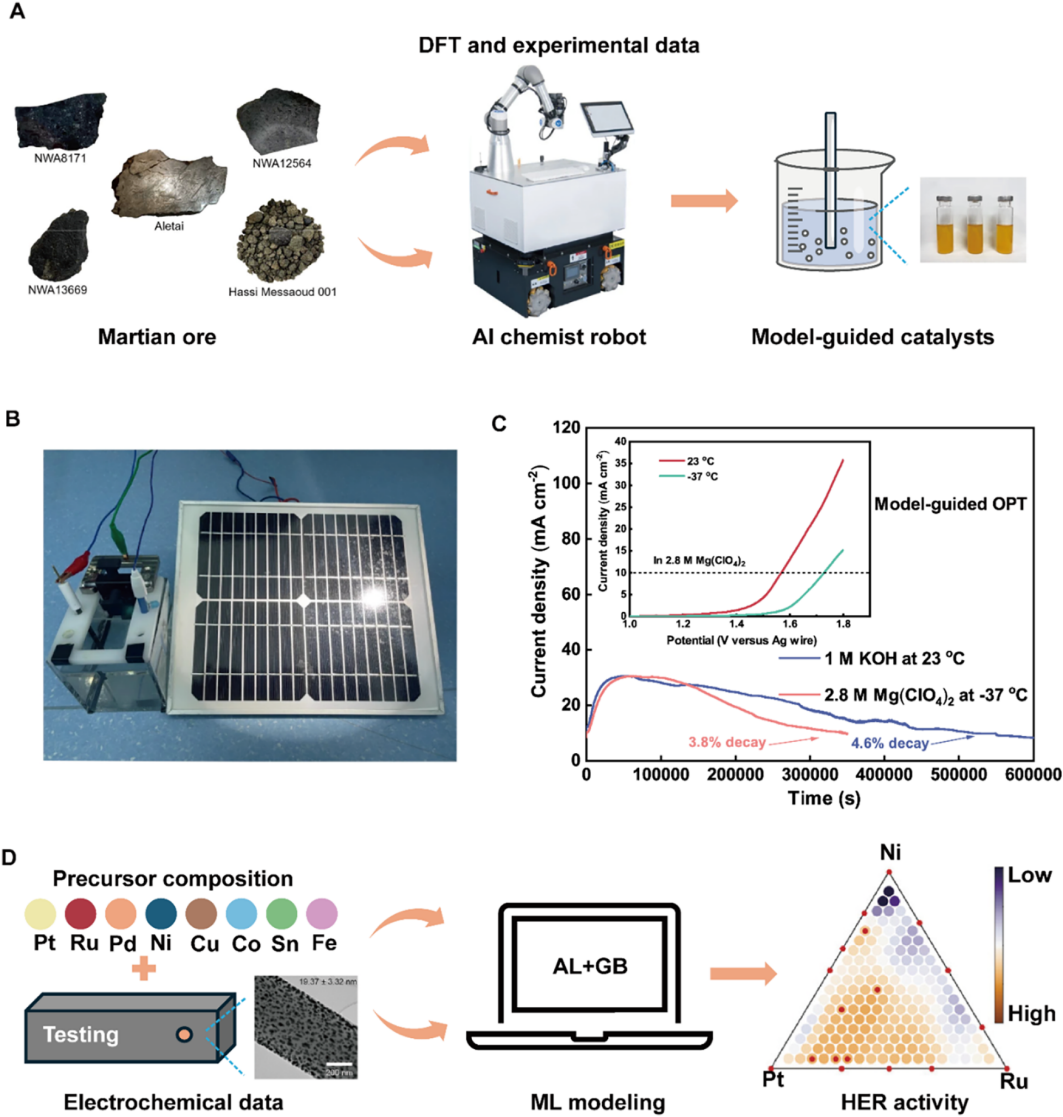
Accelerating the design of high-performance electrocatalysts. (A) Schematic diagram of fully automated AI chemist robot for screening and synthesis model-guided OPT operation. (B) Simulation of OER testing on Mars. (C) OER performance and stability in different external environments. (D) Representation for predicting HER activity using AL and GB. (A–C) Reproduced with permission from ref (^106^). Copyright 2023 Springer Nature. (D) Reproduced with permission from ref (^107^). Copyright 2022 Wiley-VCH.

Although the combination of AI and DFT is currently the most widely used and effective method for catalyst screening, it has limitations. For example, the final compositions of experimentally synthesized alloys may deviate from the stoichiometric ratios of the precursors, resulting in discrepancies between the elemental ratios observed in real experiments and those predicted by combining DFT with AI. Therefore, methods for directly training relevant AI models to guide catalyst design through experimental parameter data of material synthesis have emerged. A predictive model was built by AL combined with the GB approach using precursor mixture composition and its electrochemical data as input data. The optimal metal precursor composition of Pt_0.65_Ru_0.30_Ni_0.05_ electrocatalyst was screened out, which was successfully synthesized by the carbothermal shock method and exhibits excellent HER performance (Figure 7D).^107^ This demonstrates the advantages of AI in high-performance electrocatalysts. Different input data types can correspond to different computational models, which can ultimately guide the discovery, synthesis, and application of high-performance electrocatalysts more accurately and quickly.

### Acceleration of Electrocatalytic Performance Prediction

Overpotential and stability are two key indicators for evaluating the performance of electrocatalysts in HER, OER, and ORR. However, these two indicators often need to be evaluated by experimental measurements, incurring huge time and labor costs. The emergence of AI makes it possible to predict overpotential and stability directly. In the study of the OER/ORR performance of DACs with colossal design space, a combination of DFT and AI algorithms based on topological information with ten atomic properties descriptors as inputs, such as atomic dimensions and structure, atomic mass, atomic radius, and the number of *d* electrons, predicted the overpotential of 16,767 DACs for OER and ORR (Figure 8A,B).^17^ The results showed that 511 DACs had better OER activity than IrO_2_(110), 855 DACs had better ORR activity than Pt(111), and 248 bifunctional DACs had high catalytic performance for both OER and ORR, significantly reducing the screening time by a factor of 144,000 compared with pure DFT calculations.

**Figure 8:**
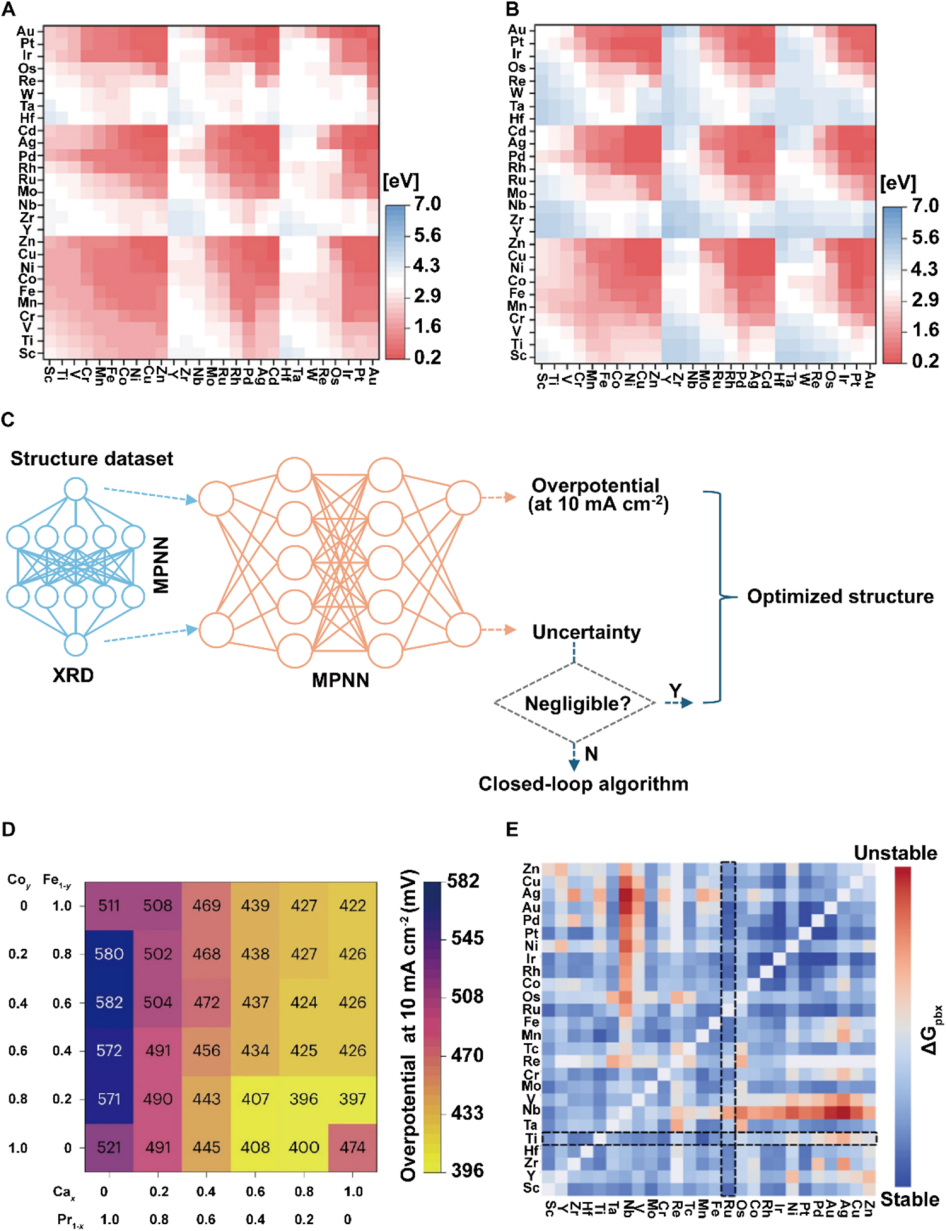
Accelerated screening of electrocatalyst activity and stability. (A) AI-predicted OER activity map for DACs catalysts. (B) AI-predicted ORR activity map for DACs catalysts. (C) Overview of combining the work of the MPNN and the closed-loop AL method. (D) The overpotential at 10 mA cm^–2^ for the oxide Ca_*x*_Pr_1–*x*_Co_*y*_Fe_1–*y*_O_3_ (0 ≤ *x*, *y* ≤ 1) is predicted based on the Ca, Pr, Co, and Fe precursor mixture ratios. (E) AI-HTS predicted heatmaps of Δ*G*_pbx_. The dashed area highlights Ru–Ti as the most stable among nonprecious metals. (A, B) Reproduced with permission from ref (^17^). Copyright 2022 Wiley-VCH. (C, D) Reproduced with permission from ref (^16^). Copyright 2023 Springer Nature. (E) Reproduced with permission from ref (^108^). Copyright 2024 American Chemical Society.

Inspired by the high correlation between the electrocatalytic performance of electrocatalytic materials and their crystal plane, the XRD generation model of perovskite oxide and the prediction model of electrocatalyst overpotential were constructed by only 30 sets of initial perovskite oxide’s crystal structure data sets and ten sets of augmented data sets combined with the MPNN framework and the closed-loop AL method (Figure 8C). The optimal OER performance of chalcogenide Ca_0.8_Pr_0.2_Co_0.8_Fe_0.2_O_3−δ_ electrocatalytic material was screened out from more than 10 thousand candidate structures (Figure 8D),^16^ and the accuracy of the prediction results was proved by successful experimental synthesis. This method demonstrates the importance of small-sample, high-quality training sets combined with AL for predicting the overpotential of high-performance electrocatalysts.

In addition, many studies use AI to predict the electrocatalytic activity of materials, but very few research works have predicted their stability. In acidic water oxidation, the stability of catalysts is highly challenged, and it is difficult for electrochemists to develop stable non-Ir electrocatalysts under acidic conditions. By training on a data set of over 3600 mixed metal oxides, an AI-HTS pipeline accurately predicted the Pourbaix decomposition energy (Δ*G*_pbx_) and facilitated screening of 2070 novel metal oxides (Figure 8E).^108^ Although Pourbaix diagrams are useful for describing the stability of metals and their oxides, it should be noted they have limitations. They assume reactions are at equilibrium, which is often not the case in real-world conditions, and they only show thermodynamic stability without considering kinetics or actual corrosion rates.^109^ Complex reaction environments and catalyst compositions further reduce their accuracy. Further, highly active electrocatalysts screened by AI do not necessarily have excellent stability, and some are prone to leaching of catalytically active sites during electrochemical processes, resulting in a significant reduction of catalytic activity.^108^ We suggest a parallel study of its stability should accompany every prediction of electrocatalytic activity. Selectivity is also a key performance indicator, particularly for reactions that yield multiple possible products, and should be considered alongside activity and stability.^14^ Finally, to ensure comparability across different studies, key testing parameters such as electrolyte composition, pH, *iR*-correction, and whether activity is reported as geometric or ECSA-normalized current should also be clearly documented.

## Perspective and Conclusion

Accelerating electrocatalyst materials discovery with AI is an emerging field with immense potential. Numerous potential research directions can further enhance the role of AI and expand the scope of applications. We broadly categorized these research directions into five areas: descriptors, data, algorithms, systems, and applications.

For ML learning models, descriptors are input features that deeply impact model performance, generalizability, and interpretability. New descriptors may improve the performance effectively. For example, in accelerating DFT calculations through NNs potentials, beyond the commonly used atom coordinates and element types, some studies have shown improvements by including the valence and spin direction as additional features.^90,110^ Recent advances in descriptor development, driven by collaboration between chemists and AI experts, along with the introduction of more informative representations such as multidimensional encodings, graph-based structures, and symmetry-aware features, have significantly enhanced the model’s ability to learn and correlate intricate chemical trends and have improved predictive power. From another perspective, ML methods can also assist in deriving high-level descriptors from simpler ones. In this regard, representation learning has been extensively studied,^111^ enabling models to autonomously extract task-relevant features from raw structural, compositional, or spectroscopic data without human intervention. These data-driven representations, when guided by specific performance objectives such as activity or selectivity, have the potential to replace conventional manually engineered descriptors and reveal underlying structure–property relationships. When applied to the discovery of electrocatalyst materials, they may offer more effective guidance for both experimental design and computational modeling. However, the development of descriptors that are both robust and widely applicable remains an unresolved challenge. Many current descriptors are highly task-specific and fail to transfer across different reaction conditions, material classes, or electrolyte environments. Symbolic regression methods such as SISSO often depend on manually selected primary features and struggle to adapt to dynamically evolving or noisy data sets.^39,40^ Furthermore, although deep learning models offer strong predictive power and are essential for capturing nonlinear descriptor interactions, the learned features often lack physical meaning, limiting their utility in mechanism-driven catalyst design. Taken together, these challenges and opportunities underscore a fundamental shift: descriptor design is no longer a peripheral technical step but a central scientific focus in intelligent electrocatalysis. While model interpretability is often viewed as a challenge intrinsic to complex simulation architectures, the selection and construction of physically meaningful descriptors can play an important supporting role by anchoring model predictions in chemically interpretable terms. In this context, interpretability should be treated as a core design objective, not only for learning algorithms but also in the representation of catalytic systems. Models must not only predict outcomes but also support scientific understanding and inference. Achieving highly interpretable, transferable, and physically consistent descriptor frameworks will require not only algorithmic innovation but also deep and sustained collaboration between AI researchers and domain experts. As NNs continue to grow in complexity, gaining a mechanistic understanding of their decision-making processes, especially how they interact with structured and physically grounded inputs, will be essential for building reliable and autonomous AI systems for catalyst discovery.

Data quality matters in training AI models. Only by collecting sufficient, high-quality data from appropriate descriptors can researchers train a satisfactory model. Currently, most electrochemical research requires collection of data from scratch. Due to various cost constraints, these task-specific data sets are often small in scale, limiting the potential of AI. Public data sets can help solve this problem, but as mentioned earlier, existing ones still need to be extended. Furthermore, results from different laboratories, equipment, and experimental methods are not always directly comparable, which further restricts data choices. To address these limitations and improve data quality, two directions are emerging: experimental data generation and computational data processing. In the experimental domain, ensuring high-quality data from manually conducted studies remains critically important. This involves the establishment of rigorous data reporting protocols, alignment of performance measurements with mechanistic study conditions, and the open sharing of raw data. These practices not only enhance the reliability of experimental results but also facilitate cross-group comparability and data reuse. Building on this foundation, standardized high-throughput platforms such as magnetron sputtering, droplet printing, and automated electrochemical testing enable parallel synthesis and evaluation of large catalyst libraries on single substrates. Automation further reduces human error and environmental noise, improving consistency and reproducibility.^112,113^ On the data side, natural language processing and web crawling are used to extract composition, structure, and performance data from literature, forming structured databases.^114^ Unified feature encoding and physically informed descriptors enhance model interpretability and enable better generalization across data sets. Closed-loop systems combining experiments, simulations, and ML enable real-time feedback and data updates, enhancing model robustness and discovery efficiency. Interdisciplinary collaboration and the development of open-access databases support standardization, data sharing, and the transition toward a data-centric research paradigm in electrochemistry. In particular, generating large-scale, high-fidelity experimental data sets under application-relevant conditions such as varying pH, electrolyte composition, and applied potentials remains a significant unmet need. Constructing task-specific databases tailored to distinct reaction types (for example, HER, OER, ORR, or CO_2_RR) or material classes (such as high-entropy alloys and SACs) could dramatically enhance the relevance and generalizability of AI models. At the same time, advances in autonomous experimentation and adaptive data acquisition through active learning will allow data sets to be continuously expanded into underrepresented regions of the compositional and operational space. The integration of these data workflows with multifidelity modeling, transfer learning, and uncertainty quantification will enable the development of more robust and generalizable models with improved data efficiency. Ultimately, progress will depend on the establishment of open, interoperable, and task-aware data infrastructures, supported by community-wide benchmarking standards and interdisciplinary collaboration, to fully realize a data-centric paradigm for intelligent catalyst discovery. In the future, establishing higher-quality data sets for a broader range of materials and tasks, especially experimental data sets,^115,116^ and better aligning data from different sources can assist in AI development and application in electrochemical studies.

AI algorithms, including models, optimizations, and improvements for specific environments, also require further refinement within electrocatalyst applications. AI models have made significant progress in recent years, from Residual Networks to Transformers, with advancements in fitting ability, generalization, explainability, and reasoning.^117,118^ However, due to limitations in data and usability, the application of novel AI models in electrocatalysis still requires further exploration. Currently, emerging generative models such as VAE, DMs, and FlowMM offer new paradigms for molecule and structure generation, yet their application in catalysis remains in its infancy.^64,68^ These models face unique challenges, including accurately capturing the multiscale physics of reactive interfaces and generating chemically valid and synthesizable candidates under electrochemical constraints. However, they require large amounts of clean, high-dimensional training data, which are rarely available in electrocatalysis. Another critical challenge is the lack of structured understanding of reaction mechanisms. The black-box nature of many deep learning models limits their reliability and adoption in catalyst discovery workflows. Moreover, existing interpretability methods, such as various feature attribution techniques, still lack strong theoretical foundations and frequently fail to rationalize abrupt changes in model outputs caused by minor perturbations in inputs. Even physics-informed ML models may exhibit instability or degraded performance in systems with incomplete or uncertain governing laws, such as proton exchange membrane electrolysis.^119^ In addition, most existing models are single-objective in nature, typically focusing on maximizing activity. Multiobjective frameworks that can simultaneously consider stability, selectivity, cost, and synthetic feasibility are still underdeveloped.^120^ Transferability across different materials systems and reaction environments is also limited, highlighting the need for more generalizable model families. Beyond these issues, model effectiveness in electrocatalysis is constrained by several factors. Current models rarely encode external stimuli such as electric fields, electrode potentials, and mechanical strain, despite their profound impact on catalytic performance. As for DFT acceleration, currently, simple models often struggle to capture the complexity of multielement systems, whereas more expressive architectures usually incur substantially higher computational overhead, which in turn impedes long-time scale simulations. Addressing this trade-off by combining model strengths, through techniques like feature extraction and knowledge distillation, remains an open challenge.^121^ Equally critical for molecular dynamics is model robustness, since a few high-error steps can destabilize a prolonged trajectory; exploring network architectures, ensemble learning, or error-correction schemes to guard against extraordinarily unexpected predictions is therefore a valuable research direction. Finally, whereas recent ML methods have begun to approximate long-range interactions, they typically require pairing with short-range potentials to form a complete force field. Developing an end-to-end framework capable of simultaneously predicting both short- and long-range forces would not only simplify the user workflow but also deepen our understanding of the underlying physics. In future, several promising directions are emerging. First, incorporating physical constraints and mechanistic priors into model design could lead to more physically consistent and interpretable predictions. Second, multimodal and multitask learning frameworks that integrate structural, spectroscopic, and electrochemical features could improve representation robustness and model generality. Third, uncertainty-aware and self-adaptive optimization strategies, such as BO or loss reweighting guided by data quality, may offer better performance under noisy or incomplete data. Fourth, transfer learning, represented by pretraining–fine-tuning paradigms, could accelerate learning in new catalytic systems. Ultimately, enhancing interpretability remains crucial. Physics-informed attention mechanisms and hybrid symbolic–neural models offer promising routes to bridge the gap between prediction accuracy and chemical insight. Collectively, advancing algorithmic foundations, together with improvements in data and descriptor design, will be key to realizing reliable, scalable, and mechanism-aware AI systems for intelligent electrocatalyst discovery.

At the system level, exploring how to implement AI algorithms for a wider range of electrocatalytic applications is crucial. While AI can significantly accelerate electrocatalyst research, it is ultimately only one node of the overall research pipeline. Optimal results can only be achieved by fully integrating AI with other software packages and tasks. Several large general models, such as ChatGPT for natural language processing and AlphaFold for protein structure prediction, have emerged in recent years.^122,123^ These models can provide predictions without requiring specific training for particular problems. However, currently they often lack domain-specific accuracy and may generate incorrect or physically inconsistent outputs. Their ability to process complex scientific data including molecular structures, spectra, and thermodynamic information is still limited.^124^ In addition, while LLM perform well within known chemical spaces, their extrapolation to new catalyst designs is not yet well understood.^125^ Future directions include developing multimodal models that integrate text, structural, and reaction data, and embedding physical principles and chemical knowledge to improve scientific reliability. Advancing lightweight, energy-efficient models will also be key to sustainable deployment at scale. With ongoing interdisciplinary progress, LLM are positioned to become central tools in rational design and accelerated discovery of next generation electrocatalysts. Better integration of existing large generative models into electrocatalyst research could lower the barrier for chemists to adopt AI tools and further enhance research efficiency. Going a step further, developing a similar model tailored for electrocatalyst materials may enable more effective use of chemically specific multimodal inputs, thereby facilitating more accurate and consistent analysis of complex electrochemical data. Beyond individual algorithmic or data set advancements, system-level orchestration of AI across the entire electrocatalysis discovery pipeline remains an open frontier. Emerging frameworks such as the Discovery and Synthesis Hub exemplify how modular machine learning components, including initial data mining, feature engineering, active learning, and domain adaptation, can be strategically integrated to guide exploration, experimentation, and validation in a closed-loop fashion.^120^ These frameworks highlight the importance of workflow modularity by enabling tailored optimization of subcomponents such as feature selection or model training, depending on specific catalyst classes or target reactions. Furthermore, as high-throughput experimentation and autonomous laboratory systems become increasingly available, AI must be capable of operating across multiple fidelity levels, spanning from theoretical simulations to experimental measurements, while reconciling divergent data standards, instrumentation outputs, and mechanistic time scales. Achieving this requires not only infrastructure standardization and software interoperability but also the development of hierarchical decision-making models that dynamically assign tasks to simulations, experiments, or surrogate models based on confidence levels and cost-efficiency. Another promising direction is task-aware orchestration, in which AI systems can prioritize candidate evaluations across competing objectives such as activity, stability, scalability, and sustainability, often under real-world operational constraints. This transition from static predictions to dynamic, multiobjective decision-making necessitates system-level frameworks that embed feedback loops throughout model inference, material synthesis, and performance evaluation.

To sum up, AI-assisted design strategies offer a new approach to discovering high-performance electrocatalyst materials. These applications include establishing predictive models by integrating DFT and AI, applying AL to acquire high-quality data with fewer experiments, utilizing interpretable algorithms, and developing fully automated chemical robotics systems combined with AI platforms.^101,102,106^ These advancements significantly reduce the time required to develop electrocatalytic materials with high activity and stability, while also accelerating the discovery of corresponding reaction mechanisms. Despite these advancements and numerous advantages of AI in assisting electrocatalyst discovery, a comprehensive understanding of the electrocatalytic reaction process necessitates advanced computer modeling, which remains challenging due to the inherent complexity of the system. Throughout the “DFT–ML–Experiment” research workflow, a pronounced mismatch and disconnect can be observed between computational predictions and experimental validations. First, while DFT has played an important role in providing thermodynamic descriptions, its simplifications of key processes—such as dynamic evolution, interfacial solvation effects, electric field influences, and surface reconstruction—often lead to theoretical predictions that fail to accurately capture the actual reaction environments.^126^ Based on such idealized data sets, ML models are unable to correct the intrinsic biases of the input and may even amplify the deviations, resulting in systematically misleading predictions.^127^ Furthermore, many current ML-accelerated studies remain at the stage of theoretical screening without systematic experimental validation, preventing the establishment of effective closed-loop feedback between computation and experiment.^126^ Even when promising candidate materials are identified, discrepancies between predicted and actual performances frequently emerge during synthesis and electrochemical testing, revealing a substantial gap between DFT modeling and real interfacial catalytic behavior. Such discrepancies are further exacerbated by the fact that experimental performance data may not reliably reflect the intrinsic activity of catalysts, as they are often influenced by system-level factors such as cell configuration, operating conditions, and measurement protocols—conditions that can differ markedly from those assumed in theoretical models. In addition, most current DFT-ML frameworks heavily rely on conventional activity descriptors, such as the d-band center and adsorption energy, along with scaling relations, which further constrain the exploration of new catalytic mechanisms and materials.^128^ These challenges fundamentally stem from the highly complex nature of electrocatalytic systems, involving the dynamic evolution of adsorption states, interfacial structural changes, fluctuations in the local reaction microenvironment, and the presence of surface defects, all of which greatly complicate accurate modeling and reliable prediction. To enhance the overall effectiveness of the workflow, future efforts should aim to extend DFT modeling toward realistic operating conditions (operando environments), strengthen the coupling of multiphysical processes such as solvent effects, electric field interactions, and interfacial dynamics, and establish rigorous two-way experimental–theoretical validation frameworks, while introducing uncertainty quantification and dynamic learning strategies to improve the reliability and generalizability of catalyst discovery. Importantly, achieving a profound understanding of electrocatalytic mechanisms and realizing breakthrough material designs cannot solely rely on computational chemistry or AI. A collaborative approach involving theoretical chemists, computer scientists, and experimental chemists will be crucial to advancing this field, while future progress will also depend on developing transferable and interpretable descriptors and building reliable experimental databases to complement computational resources. Together, these efforts can establish a more robust and data-driven paradigm for the rational discovery of next-generation electrocatalysts.
